# Association of the NAD(P)H oxidase p22 phox gene C242T polymorphism with type 2 diabetes mellitus, diabetic nephropathy, and carotid atherosclerosis with type 2 diabetes mellitus: A meta-analysis

**DOI:** 10.1016/j.mgene.2015.07.009

**Published:** 2015-08-25

**Authors:** Tao Li, Hai-feng Xi, Hong-min Luo, Wen-xuan Liu, Xia Gao, Dian-wu Liu, Lei Yang

**Affiliations:** a Department of Epidemiology and Statistic, Hebei Medical University, Shijiazhuang 050017, China; b Hebei Provincial Health and Family Planning Commission, Shijiazhuang, China; c Department of Nephrology Third Hospital, Hebei Medical University, Shijiazhuang, China

**Keywords:** Type 2 diabetes mellitus, Diabetic nephropathy, Carotid atherosclerosis, Polymorphism, p22 phox

## Abstract

**Background:**

Several epidemiological studies have evaluated the association between the NAD(P)H oxidase p22 phox gene C242T polymorphism and the risk of type 2 diabetes mellitus (T2DM), diabetic nephropathy (DN), and carotid atherosclerosis with T2DM (CA), but the results are inconclusive. This meta-analysis was therefore designed to clarify these controversies.

**Methods:**

Systematic searches were performed using electronic databases such as MEDLINE, PubMed, EMBASE, and China National Knowledge Infrastructure, as well as through manual searching of the references of identified articles. A total of 11 publications were eligible for this meta-analysis after running a search on the NAD(P)H oxidase p22 phox gene C242T polymorphism, including 7 with outcomes for T2DM, 7 with outcomes for DN, and 3 with outcomes for CA. The pooled odds ratio (OR) with a 95% confidence interval (CI) was calculated using a fixed effects model (FEM) or a random effects model (REM). Publication bias was tested by Begg's funnel plot analysis. Sensitivity analysis was also performed.

**Results:**

The results showed a significant association between the NAD(P)H oxidase p22 phox gene C242T polymorphism and T2DM risk in the allelic model (REM: OR = 1.23, 95% CI = 1.06–1.43), additive model (FEM: OR = 1.61, 95% CI = 1.14–2.26), and recessive model (FEM: OR = 1.50, 95% CI = 1.10–2.05). A significant association was also observed for DN in the allelic model (REM: OR = 1.25, 95% CI = 1.06–1.47), additive model (FEM: OR = 1.61, 95% CI = 1.08–2.38), and dominant model (REM: OR = 1.26, 95% CI = 1.03–1.54). However, no association was observed for CA. Similar results were obtained in subgroup analysis based on ethnicity.

**Conclusions:**

Results of this meta-analysis suggest that the NAD(P)H oxidase p22 phox gene 242T allele might be associated with an increased risk of T2DM and DN, but not CA.

## Highlights

• There are significant association between P22 phox gene C242T polymorphism with T2DM DN risk but no significant association with CA.

## Graphical abstract

**Figure:**
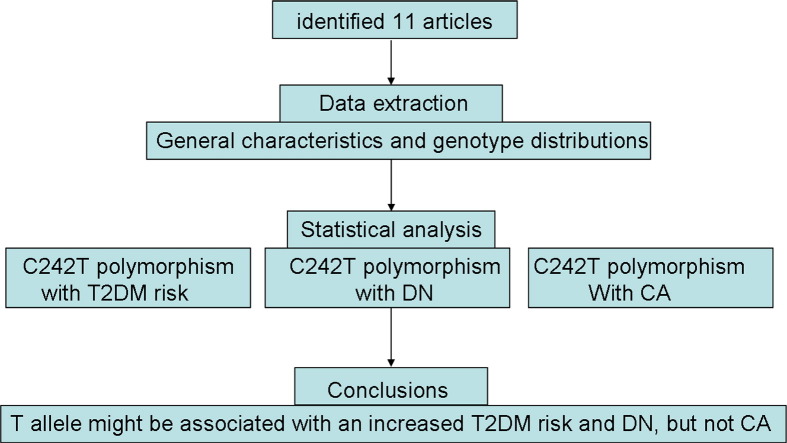

## 1 Introduction

Type 2 diabetes mellitus (T2DM) is one of the most common chronic diseases, causing significant morbidity and mortality worldwide (Gulliford and Charlton, 2009). Patients in whom diabetes is not efficiently controlled have a significantly higher risk of developing complications such as cardiovascular disease, chronic kidney failure, retinal damage, and nerve damage (Abbas et al., 2013a). For example, hyperglycemia induces a large number of alterations in vascular tissue that potentially promote accelerated atherosclerosis (Farrag and Eid, 2008). Diabetic nephropathy (DN) is the main cause of end-stage renal disease, which is associated with cardiovascular disease and increases mortality in subjects with type 2 diabetes mellitus (Duran-Salgado and Rubio-Guerra, 2014). The rising prevalence of T2DM and the importance of early detection and management have led many investigators to search for environmental and genetic risk factors for T2DM and T2DM-related complications (Florez, 2008; Hosseini et al. 2015; Makuc and Petrovic, 2011; Moreno and Fuster, 2004; Starcevic and Petrovic, 2013).

It is well known that oxidative stress plays an important role in the development of T2DM and its complications (Opara, 2004). Hyperglycemia exaggerates O^2 −^ production from the mitochondrial respiration process with consequential activation of NADPH oxidase and redox sensitive signaling pathways implicated in diabetic complications (Gao and Mann, 2009). NAD(P)H oxidase is one of the major sources of superoxide production in vascular cells. This is a multisubunit enzyme complex and the p22 phox subunit is required for its oxidase activity (Azumi et al., 1999). The p22 phox gene is encoded by the CYBA gene, which is located on the long arm of chromosome 16 at position 24 (Dinauer, 1990) and is essential for the activation of NAD(P)H oxidase (Ushio-Fukai et al. 2002). Several polymorphisms in the p22 phox gene have been reported to modulate NAD(P)H oxidase activity. One of these, the C242T polymorphism (His72Tyr), is located on a potential heme-binding site; it is found at exon 4 of the CYBA gene (16q24), encoding the p22 phox subunit of NAD(P)H oxidase (Dinauer, 1990). A variety of epidemiological studies have evaluated the role of the NAD(P)H oxidase p22 phox gene C242T polymorphism in relation to T2DM, DN and carotid atherosclerosis with T2DM (CA), but results are inconclusive (Gu et al. 2013; Lim et al. 2006; Matsunaga-Irie et al. 2004; Narne et al. 2012; Narne et al. 2014). The present meta-analysis was therefore designed to more precisely estimate the association between the NAD(P)H oxidase p22 phox gene C242T polymorphism and T2DM, DN and CA.

## 2 Methods

### 2.1 Literature search

This meta-analysis followed the Preferred Reporting Items for Systematic Reviews and Meta-analyses criteria. The MEDLINE, PUBMED, EMBASE, China National Knowledge Infrastructure, and Web of Science databases were searched (the last search was conducted before February 10, 2015) using the following search terms: (‘NAD(P)H oxidase’ OR ‘p22 phox’ OR ‘CYBA’) AND (‘polymorphism’ OR ‘mutation’ OR ‘variant’ OR ‘genotype’) AND (‘diabetes mellitus’ OR ‘DM’). The relevant articles, texts, and reference lists were manually searched to broaden the scope of our findings. The search was limited to English and Chinese language papers and human subject studies.

### 2.2 Inclusion and exclusion criteria

The following studies were included: case–control studies that evaluated the association between the NAD(P)H oxidase p22 phox gene polymorphism and the conditions T2DM, DN and CA; studies with full text articles; and studies with sufficient genotype data presented to calculate the odds ratios (OR) and 95% confidence interval (CI). Studies deviating from the Hardy–Weinberg equilibrium (HWE) were not removed.

The following studies were excluded: reviews, abstracts, studies with insufficient information for data extraction, or studies that focused on subjects with type 1 diabetes mellitus.

### 2.3 Data extraction

Information was carefully extracted from all eligible publications independently by two authors. Discrepancies were adjudicated by a third reviewer until consensus was achieved on every item. If the same data appeared in different studies, the result was only used once. The following items were collected from each study: (1) characteristics of study participants; (2) case and control definitions; and (3) genetic data (including allelic distribution and genotypic frequency).

### 2.4 Statistical analysis

Stata 12.0 statistical software was used to perform the meta-analysis. The gene–disease association was determined by calculating odds ratios (ORs) with 95% confidence interval (CI) under the allelic model (T allele vs. C allele), additive model (TT vs. CC), recessive model (TT vs. TC + CC), and dominant model (TT + TC vs. CC). Heterogeneity among studies was assessed using the Q-test with the inconsistency index (I^2^) statistic interpreted as the proportion of total variation contributed by between-study variation (Higgins et al. 2003). If substantial heterogeneity was present (I^2^ > 50%), the DerSimonian and Laird random effects model (REM) was adopted as the pooling method; otherwise, the fixed effects model (FEM) was used as the pooling method.

To validate the credibility of the meta-analysis outcomes, a sensitivity analysis was carried out based on HWE (studies with HWE were included) and mostly odds ratios (OR ˂ 3) (studies with outlier OR > 3 were included). Begg's funnel plot (the funnel plot should be asymmetric when there is publication bias or symmetric in the case of no publication bias) and Egger's regression test (P < 0.05 was considered representative of statistically significant publication bias) were used to assess publication bias (Egger et al. 1997). In addition, subgroup analysis by ethnicity was also performed.

## 3 Results

### 3.1 Characteristics of studies

**Fig. 1:**
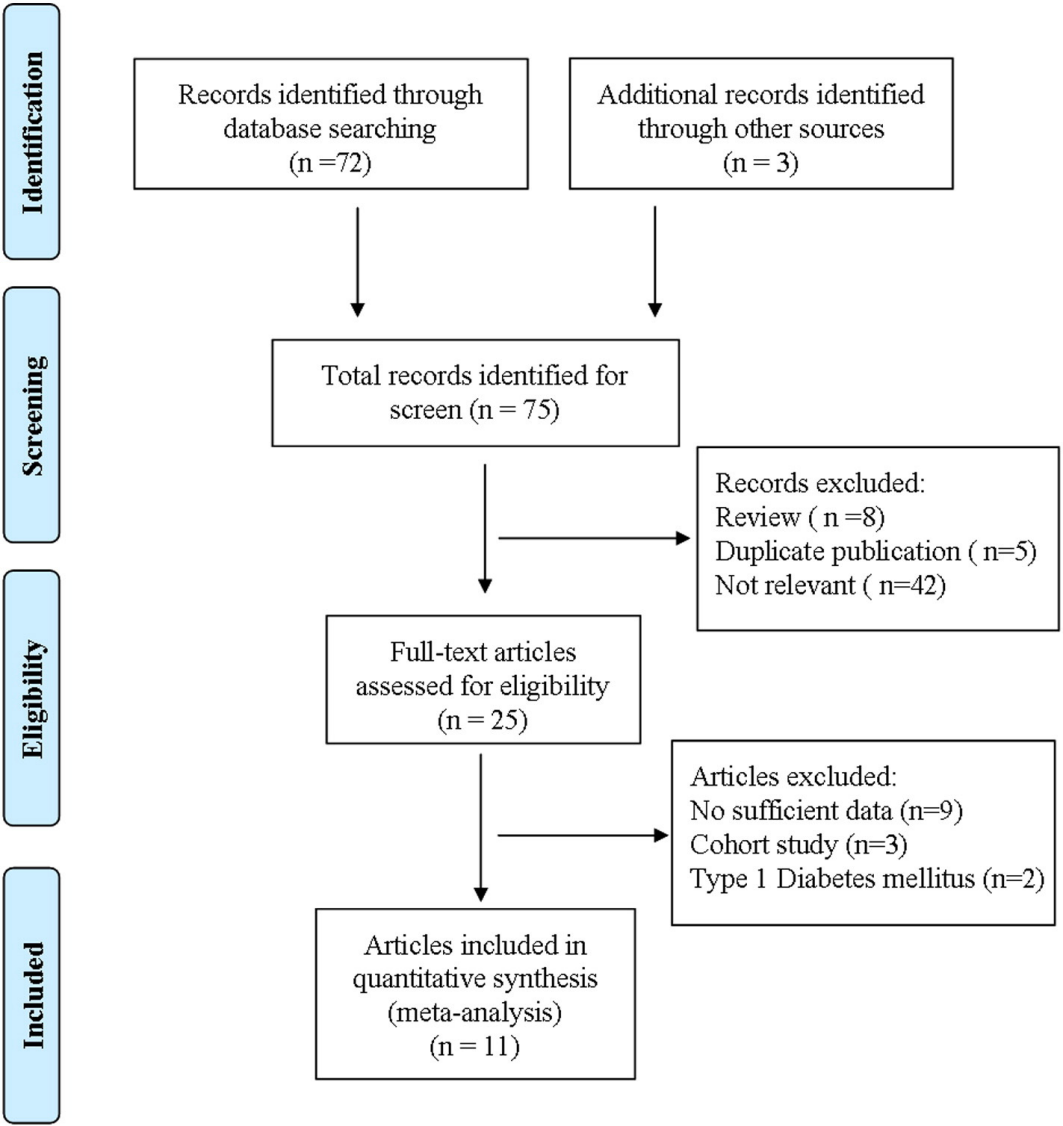
Flowchart of the literature selection process.

Based on our preliminary search criteria, we identified 75 articles during our initial electronic search. In order to assess the appropriateness of the articles, their article abstracts were screened. Fifty articles (review articles, duplicate publications, and articles that were not relevant) were excluded during the initial review. We retrieved the full text for the remaining 25 articles for further evaluation using the inclusion criteria, and a total of 11 publications were found eligible for this meta-analysis (Doi et al. 2005; Hayaishi-Okano et al. 2003; Jin et al., 2011; Letonja et al. 2012; Lim et al. 2006; Liu et al., 2006; Matsunaga-Irie et al. 2004; Narne et al. 2014; Santos et al., 2005; Yan, 2005; Yang et al., 2006), including 7 with outcomes for T2DM, 7 with outcomes for DN, and 3 with outcomes for CA resulting from a search on the NAD(P)H oxidase p22 phox gene C242T polymorphism (Fig. 1). All 11 articles had a case–control design. The Newcastle-Ottawa-Scale (NOS) scores of all included studies were ≥ 5 (data not show). General characteristics and genotype distributions of the above-mentioned three types of associations (T2DM, DN and CA) in the 11 studies are shown in Table 1.

### 3.2 Results of the meta-analysis

#### 3.2.1 The p22 phox gene C242T polymorphism and T2DM

**Fig. 2:**
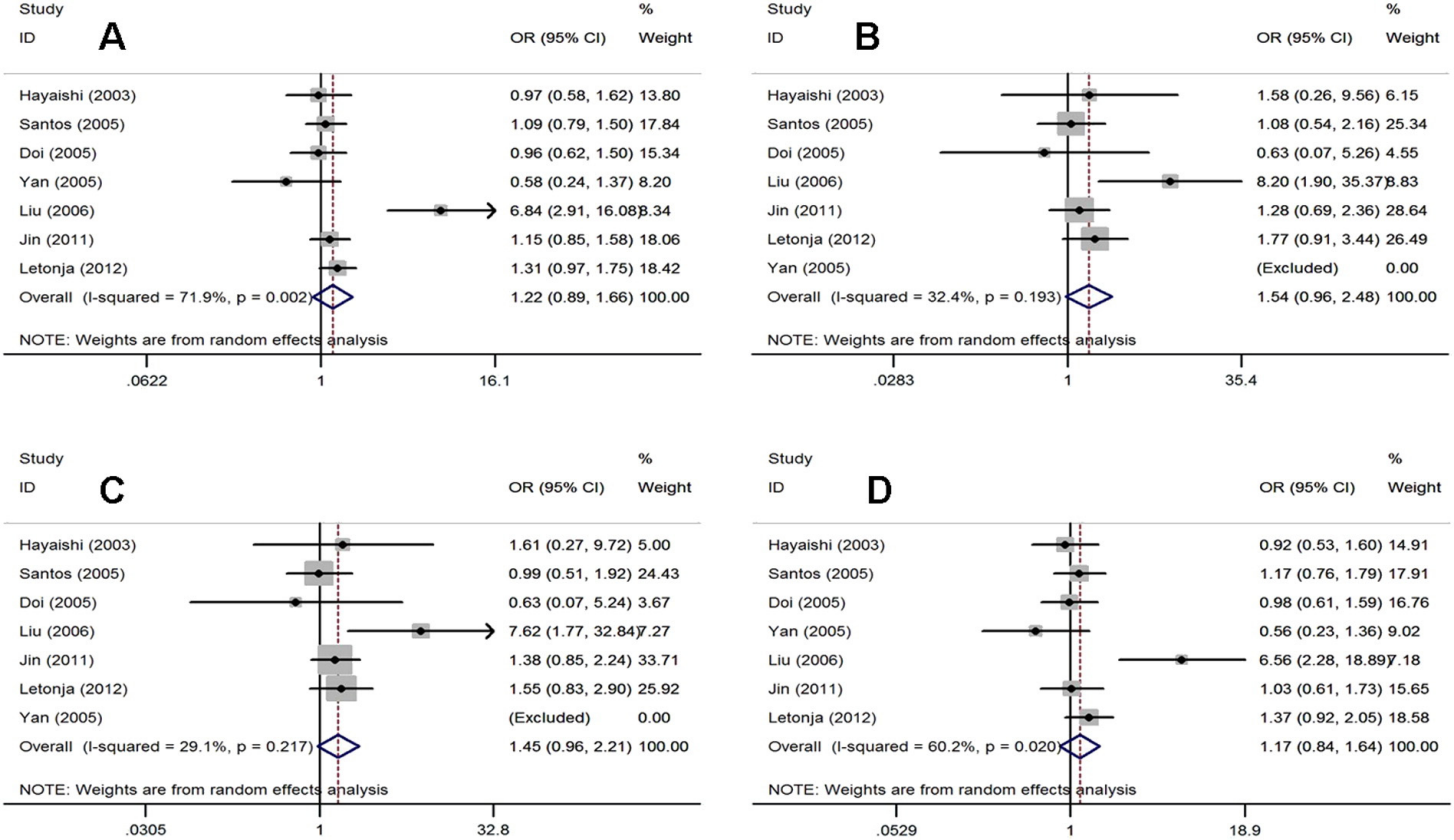
Forest plots for the p22 phox gene C242T polymorphism and T2DM risk. A. allelic model: T allele vs. C allele; B. additive model: TT vs.CC; C. recessive model: TT vs. TC + CC; D. dominant model: TT + T/C vs. C/C.

As shown in Fig. 2 and Table 1, a total of 1661 cases (patients with T2DM) and 1265 controls (healthy subjects) were identified for analysis of the association between the NAD(P)H oxidase p22 phox gene C242T polymorphism and T2DM risk.

The fixed effects model (FEM) was used for the additive and recessive models (I^2^ = 32.4%, P = 0.193; I^2^ = 29.1%, P = 0.217, respectively) and the random effects model (REM) was used for the allelic and dominant models (I^2^ = 71.9%, P = 0.002; I^2^ = 61.2%, P = 0.020, respectively). Overall, the NAD(P)H oxidase p22 phox gene C242T polymorphism was significantly associated with T2DM risk in the allelic model (REM: OR = 1.23, 95% CI = 1.06–1.43), additive model (FEM: OR = 1.61, 95% CI = 1.14–2.26), and recessive model (FEM: OR = 1.50, 95% CI = 1.10–2.05), but not in the dominant model (REM: OR = 1.20, 95% CI = 0.99–1.46) (Fig. 2). In the subgroup analyses by ethnicity, a significant association was found only for Asians between the NAD(P)H oxidase p22 phox gene C242T polymorphism and T2DM risk (recessive model, FEM: OR = 1.74, 95% CI = 1.15–2.64) (Table 2).

#### 3.2.2 The p22 phox gene C242T polymorphism and DN

**Fig. 3:**
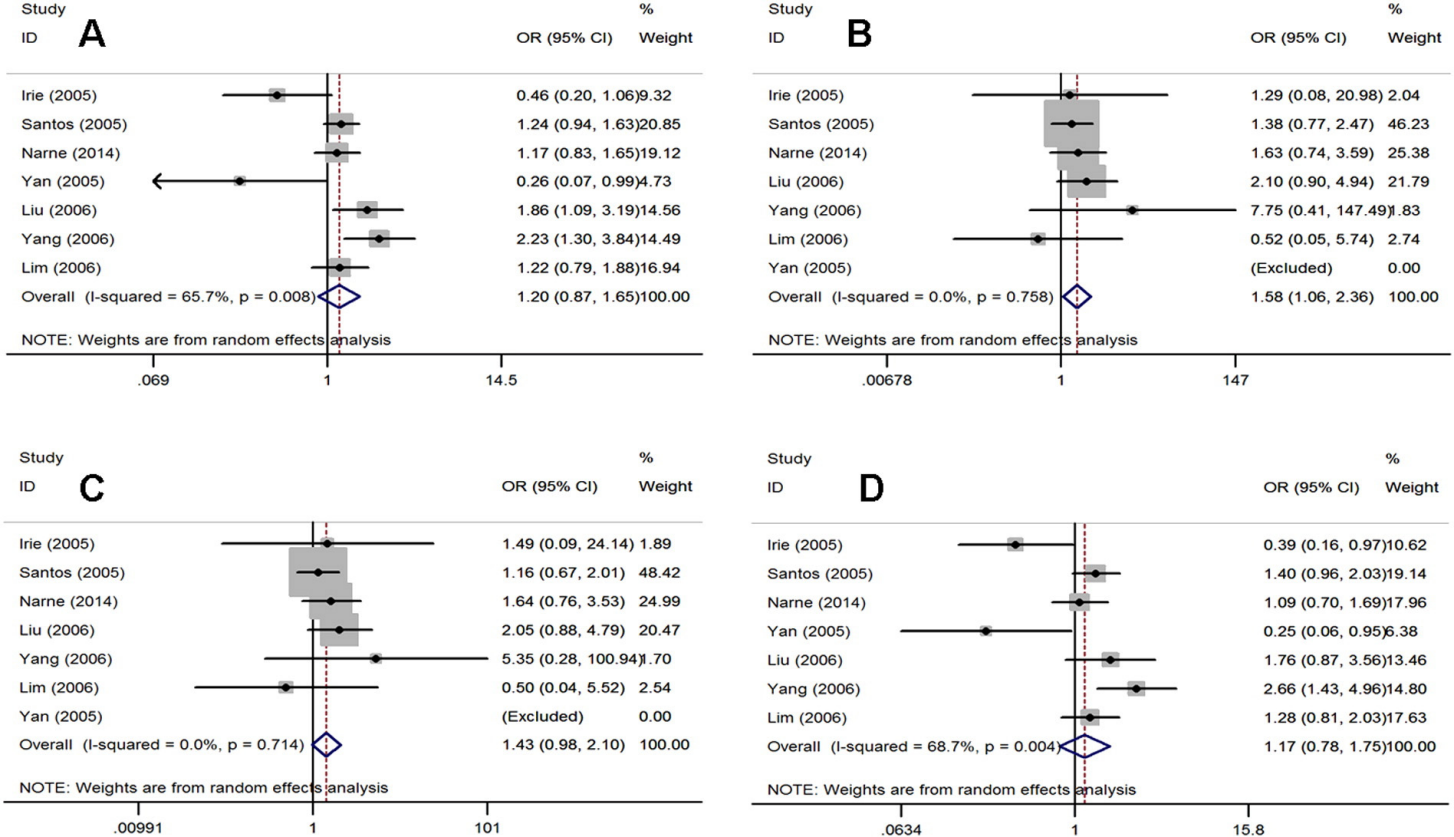
Forest plots for the p22 phox gene C242T polymorphism and DN risk. A. allelic model: T allele vs. C allele; B. additive model: TT vs.CC; C. recessive model: TT vs. TC + CC; D. dominant model: TT + T/C vs. C/C.

As shown in Fig. 3 and Table 1, a total of 1068 cases (T2DM patients with DN) and 1026 controls (T2DM patients without DN) were identified for the analysis of the association between the p22 phox gene C242T polymorphism and DN.

The FEM was used for the additive and recessive models (I^2^ = 0.0%, P = 0.758; I^2^ = 0.0%, P = 0.714, respectively) and the REM was used for the allelic and dominant models (I^2^ = 65.7%, P = 0.008; I^2^ = 68.7%, P = 0.004, respectively). Overall, the NAD(P)H oxidase p22 phox gene C242T polymorphism was significantly associated with DN risk in the allelic model (REM: OR = 1.25, 95% CI = 1.06–1.47), additive model (FEM: OR = 1.61, 95% CI = 1.08–2.38), and dominant model (REM: OR = 1.26, 95% CI = 1.03–1.54) (Fig. 3), but not in the recessive model (FEM: OR = 1.45, 95% CI = 0.99–2.12). In the subgroup analyses by ethnicity, a significant association was found only for Asians between the NAD(P)H oxidase p22 phox gene C242T polymorphism and DN risk in the additive model (FEM: OR = 1.78, 95% CI = 1.04–3.07) and recessive model (FEM: OR = 1.75, 95% CI = 1.03–2.99) (Table 2).

#### 3.2.3 The p22 phox gene C242T polymorphism and CA

**Fig. 4:**
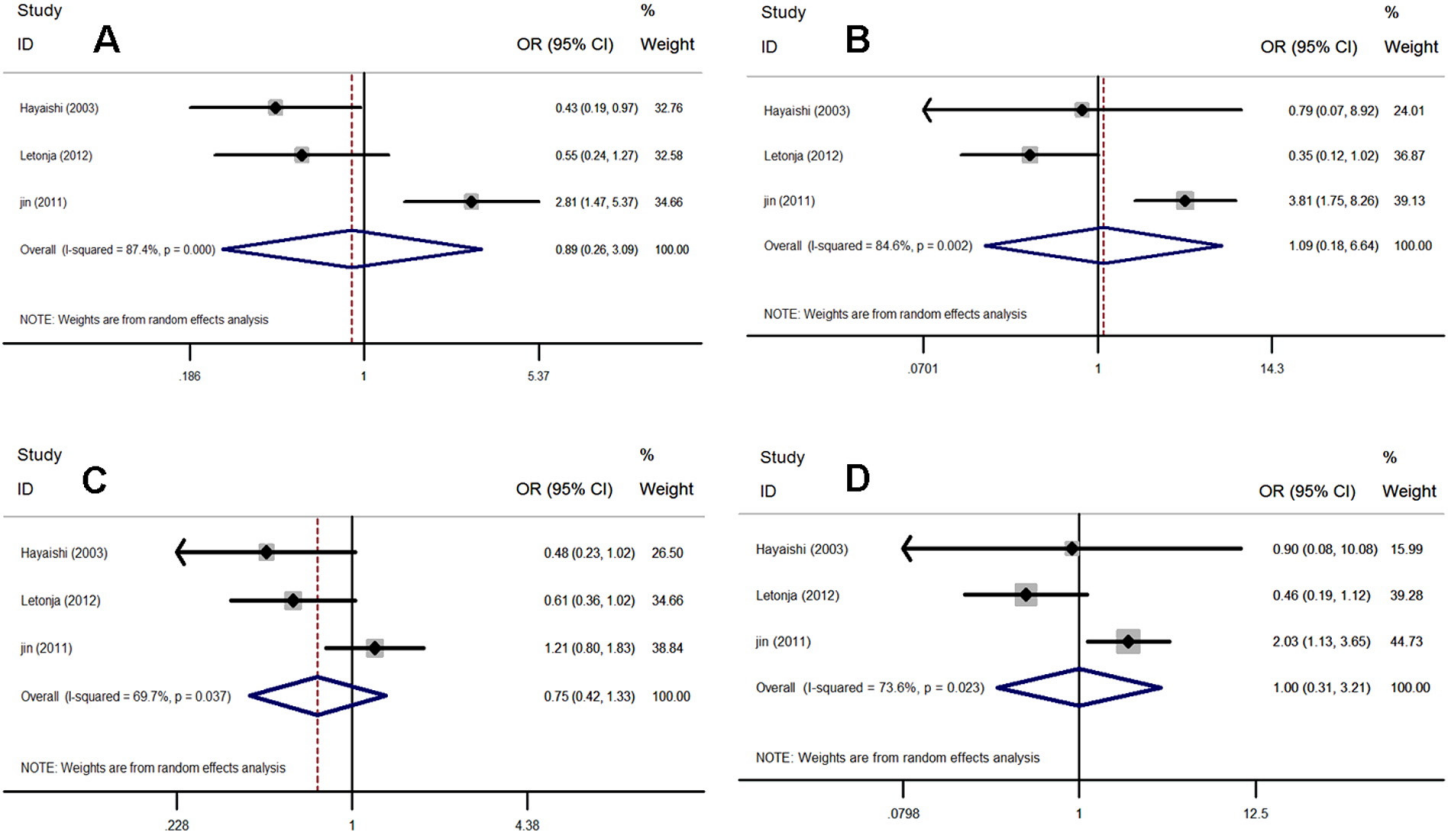
Forest plots for the p22 phox gene C242T polymorphism and CA risk. A. allelic model: T allele vs. C allele; B. additive model: TT vs.CC; C. recessive model: TT vs. TC + CC; D. dominant model: TT + T/C vs. C/C.

As shown in Fig. 4 and Table 1, a total of 386 cases (T2DM patients with CA) and 273 controls (T2DM patients without CA) were identified for the analysis of the association between the p22 phox gene C242T polymorphism and CA. The REM was adopted for all genetic models (I^2^ = 87.4%, P = 0.000; I^2^ = 84.6%, P = 0.002; I^2^ = 69.7%, P = 0.0037; I^2^ = 73.6%, P = 0.023, respectively). Overall, the NAD(P)H oxidase p22 phox gene C242T polymorphism was not associated with CA risk in the allelic model (REM: OR = 0.85, 95% CI = 0.63–1.14), additive model (REM: OR = 1.61, 95% CI = 0.91–2.88), recessive model (REM: OR = 1.30, 95% CI = 0.80–2.12), and dominant model (REM: OR = 1.13, 95% CI = 0.75–1.69).

### 3.3 Sensitivity analysis

Sensitivity was analyzed in two ways: by limiting the meta-analysis to studies conforming to the HWE (P > 0.05) or mostly odd ratios (OR ˂ 3). In our meta-analysis, the article without HWE (p ˂ 0.05) and that with outlier OR values (OR > 3) was the same article, so only Liu's study was excluded from the sensitivity analysis for T2DM risk and for DN risk (Liu et al., 2006). We also found that the OR and 95% CI were materially altered by limiting the meta-analysis to studies conforming to the HWE or those with outlier OR values, and the risk effects of the T allele were no longer significant. Thus, the study without HWE or with outlier OR values should be considered as a factor influencing the overall results. The results of the sensitivity analysis are shown in Table 2.

### 3.4 Publication bias

**Fig. 5:**
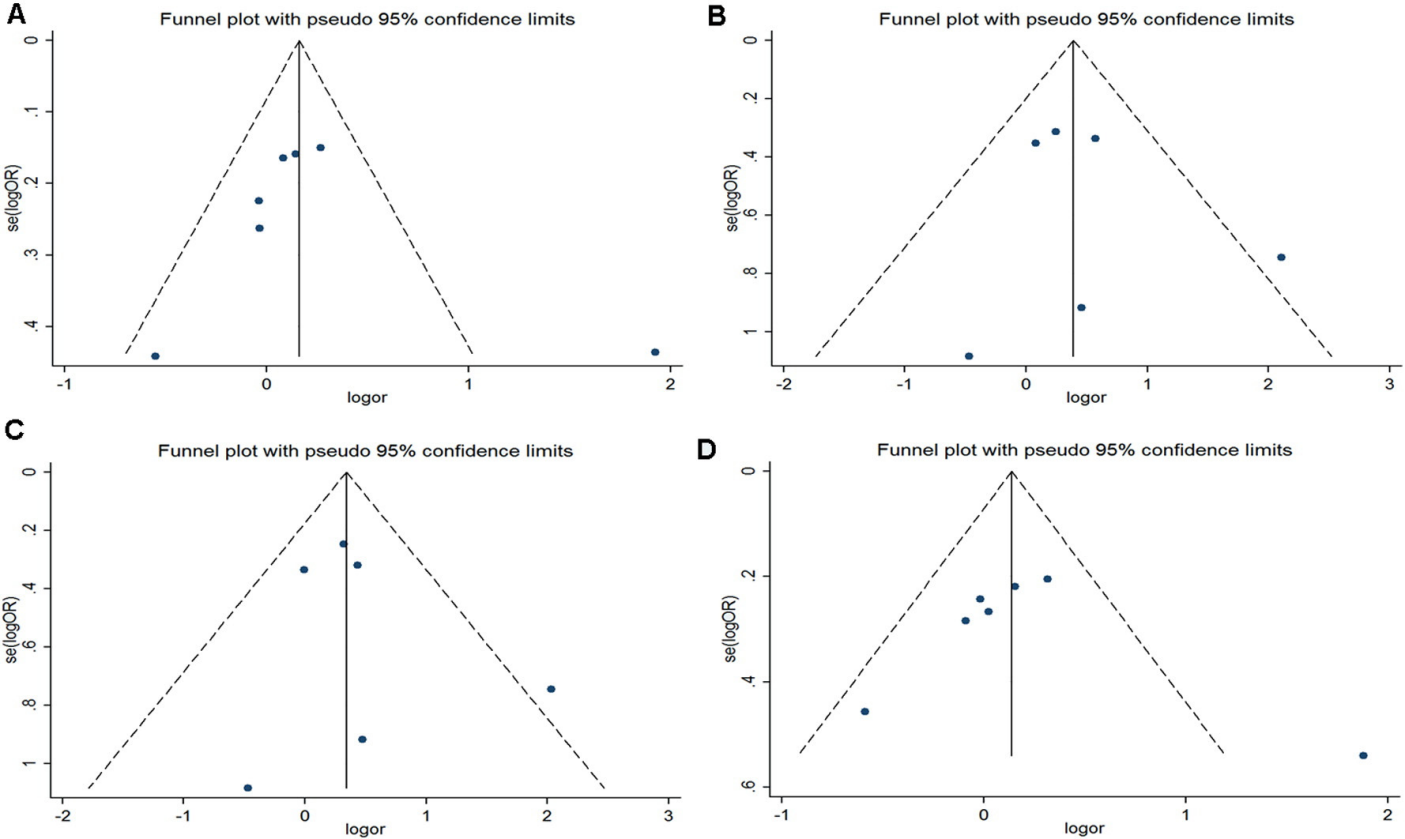
Funnel plots for the p22 phox gene C242T polymorphism and T2DM risk. A. allelic model: T allele vs. C allele; B. additive model: TT vs.CC; C. recessive model: TT vs. TC + CC; D. dominant model: TT + T/C vs. C/C.

**Fig. 6:**
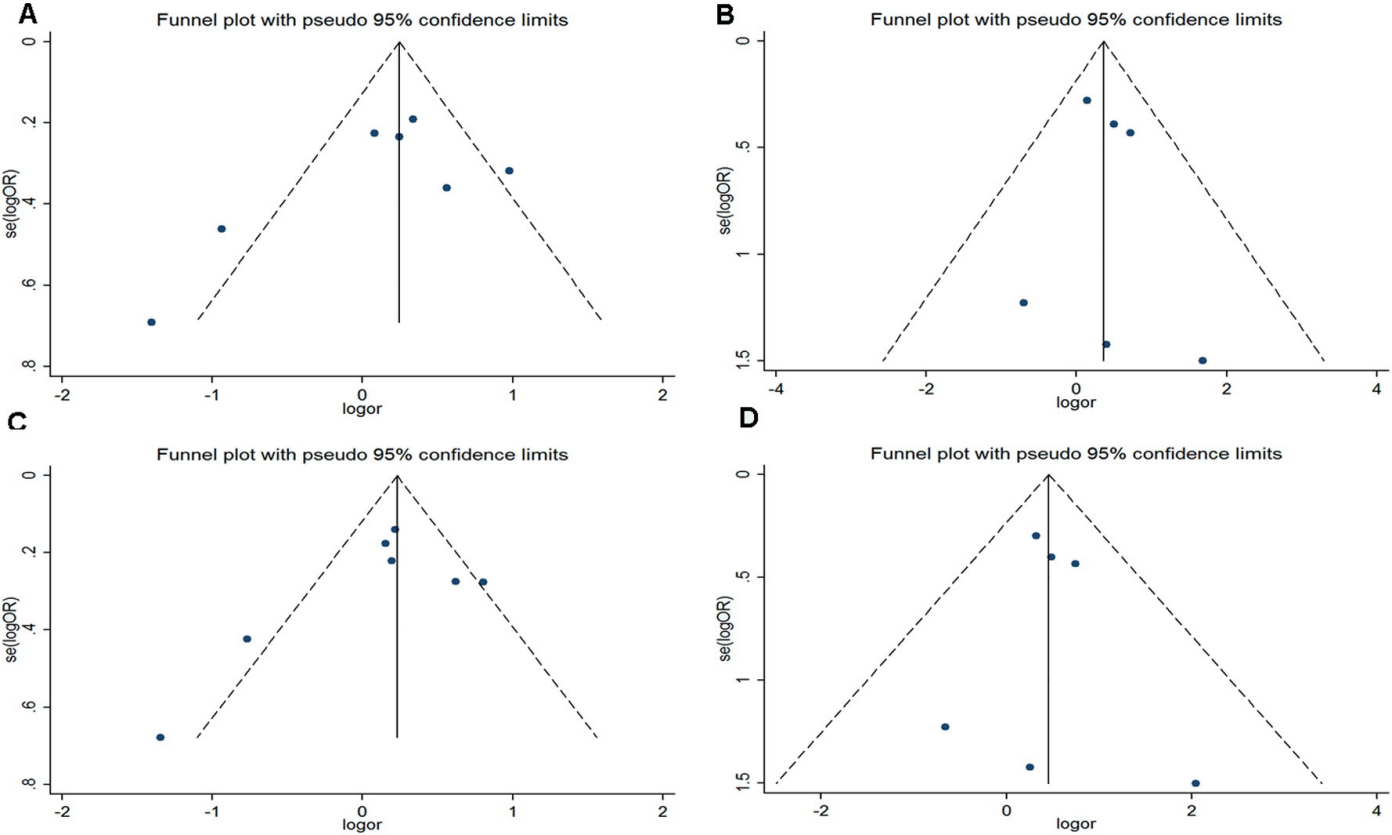
Funnel plots for the p22 phox gene C242T polymorphism and DN risk. A. allelic model: T allele vs. C allele; B. additive model: TT vs.CC; C. recessive model: TT vs. TC + CC; D. dominant model: TT + T/C vs. C/C.

**Fig. 7:**
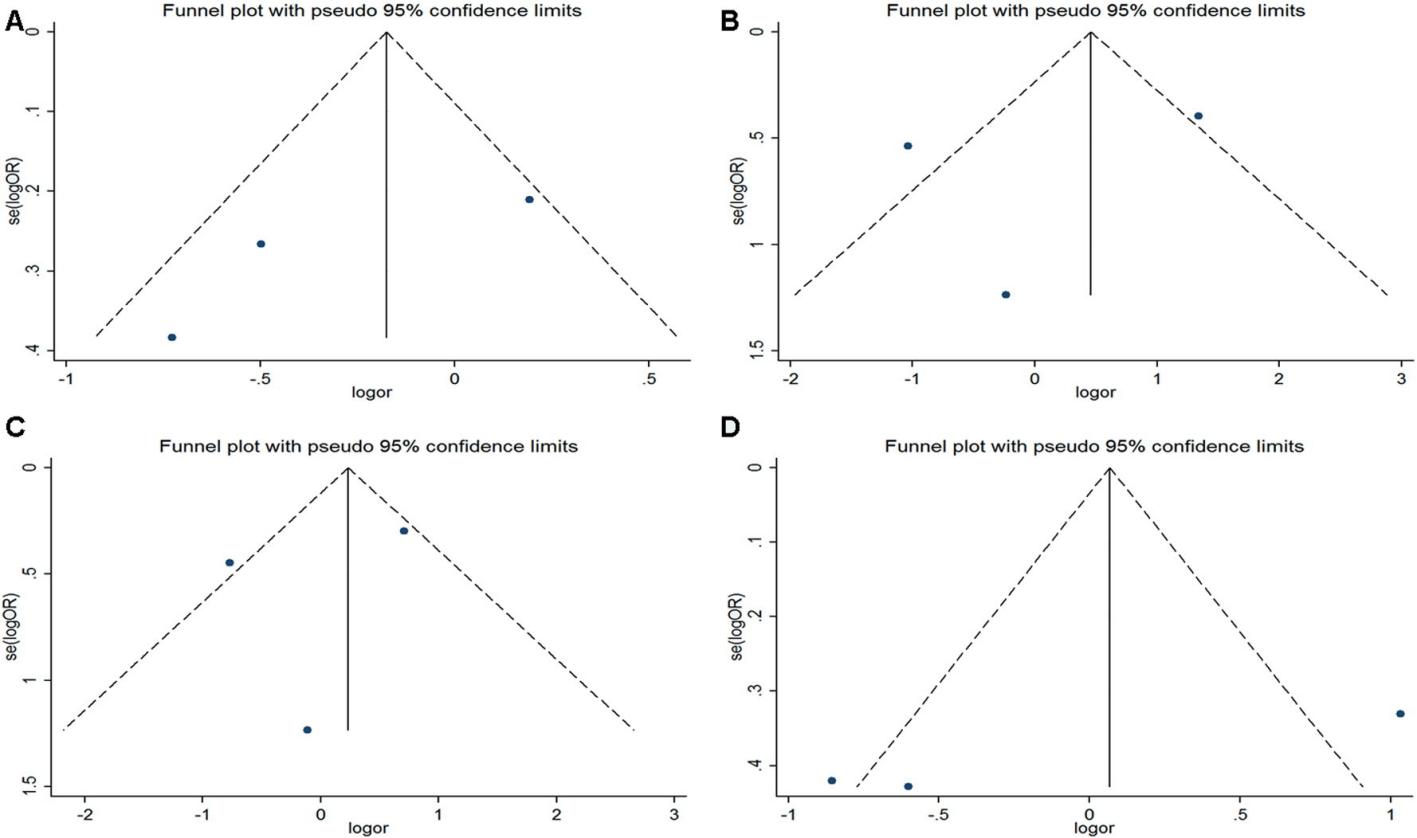
Funnel plots for the p22 phox gene C242T polymorphism and CA risk. A. allelic model: T allele vs. C allele; B. additive model: TT vs.CC; C. recessive model: TT vs. TC + CC; D. dominant model: TT + T/C vs. C/C.

In this meta-analysis, Begg's funnel plot and Egger's regression test were used to assess publication bias. In Begg's funnel plot analysis, the funnel plots did not seem to be asymmetric (Figs. 5–7). In addition, the results of Egger's regression test did not provide any evidence of publication bias. Thus, the results of this meta-analysis are relatively stable and publication bias is unlikely to have affected the results.

## 4 Discussion

T2DM and its complications are multifactorial, leading to high morbidity and mortality. Increasing evidence from evidence-based studies supports the critical role of genetic factors in the development of T2DM and its complications (Hosseini et al. 2015; Makuc and Petrovic, 2011; Starcevic and Petrovic, 2013). Oxidative stress plays a central role in the development of T2DM (Tangvarasittichai, 2015; Zephy and Ahmad, 2015). Among the oxidative stress genes, the role of NAD(P)H oxidase p22 phox has garnered attention (Fang et al. 2010; Li et al., 2013b; Li et al. 2015). Here, we focused on the association between the NAD(P)H oxidase p22 phox gene C242T polymorphism and T2DM and its complications.

In this meta-analysis, we first investigated the association between the NAD(P)H oxidase p22 phox gene C242T polymorphism and T2DM risk, and found the T allele to be associated with an increased risk of T2DM. This is consistent with the results of Sun's meta-analysis (Sun et al. 2014) but differs from the results of Xu's study, which indicate that the T allele plays a protective role in CAD (Xu et al. 2014). Then we analyzed the association between the C242T polymorphism and the risk of DN and CA; we found that the T allele was associated with an increased risk of DN, but there was no association between the T allele and CA. Variation in disease pathogenesis and inadequate sample size of research studies may be responsible for the inconsistent results.

Caution should be taken when interpreting our results on the associations of gene polymorphisms with diseases, considering the mixed ethnic composition of subjects. This is why we also performed subgroup analysis by ethnicity. The results of subgroup analysis still show a significant association between the p22 phox gene polymorphism and T2DM and DN. However, with only one or two studies included in the non-Asian subgroup determining the association of the p22 phox gene polymorphism with T2DM and DN risk, we cannot exclude the possibility that the lack of an association in non-Asian and Asian subjects might be due to the limited number of studies and the consequent lack of statistical power. Moreover, since there were only three studies related to the p22 phox gene C242T polymorphism and CA, we could not analyze the association by ethnicity for this complication.

Meanwhile, heterogeneity was apparent in the meta-analysis, with a wide range of potential sources, such as design quality, non-comparable measures of genotyping, and variations in the covariates. On aiming to clarify the sources of heterogeneity, we conducted sensitivity analyses by limiting the studies to those conforming to the HWE or with outlier OR values, which can validate the credibility of outcomes. Deviations from the HWE in control subjects may bias the estimates of genetic effects in genetic association studies and meta-analyses, so studies that appear to deviate from the HWE should be investigated further for weaknesses in their design; and these studies should not be excluded unless there are other grounds for doubting their quality (Minelli et al. 2008). In the meta-analysis, we found that the corresponding pooled ORs were materially altered for T2DM and DN, on limiting the meta-analysis to studies conforming to the HWE or without outlier OR values (OR > 3). This indicates that the study not conforming to the HWE or with outlier OR values (OR > 3) should be considered a factor that influenced the overall results. Therefore, heterogeneity might result from differences related to ethnicity or nonconformance to the HWE. However, given that heterogeneity had a wide range of potential sources, we assumed that all the potential reasons mentioned above were taken into account.

Meta-analyses are increasingly used to help make clinical decisions because pooling data from a number of studies reduces both bias and uncertainty. However, the conclusion may be affected by a type of selection bias caused by rapidly published studies with statistical results. In this meta-analysis, a funnel plot was used to determine the presence of publication bias. The plot was symmetrical, suggesting no evidence of publication bias among the studies.

The current meta-analysis has several limitations that may affect the conclusions. First, only published data from the selected databases were included, causing us to miss eligible studies that may have been unpublished or unreported, studies in other languages, and master's theses. Although no significant publication bias was detected in the meta-analysis, it should be recognized that publication bias is indeed difficult to exclude. Second, the sample size for the analyses was small, although the results are positive. In particular, the subgroup analyses were based on only two ethnicities, so additional studies need to be collected for further analysis in the future. Third, the gene-gene or gene-environment interaction may influence the association between the p22 phox gene C242T polymorphism and T2DM, DN, and CA. However, no appropriate data are available from reported studies. Finally, although available genetic data suggest that the p22 phox gene C242T polymorphism is a determinant of T2DM and DN susceptibility, the corresponding pooled ORs were materially altered in sensitivity analyses. Thus, studies that appear to deviate from the HWE should be investigated further for weaknesses.

To conclude, the present meta-analysis suggests that the NAD(P)H oxidase p22 phox gene 242T allele might be associated with an increased risk of T2DM and DN, but not CA. However, based on limited data from published studies, it is difficult to draw definitive conclusions. Owing to the aforementioned limitations and the variations in the risk of T2DM and its complications, further investigations will be needed to confirm our findings.

## Funding

The authors declare no conflict of interest.

## Figures and Tables

**Table 1:** Characteristics of studies included in this meta-analysis.

First author	Year	Country	Ethnicity	Case (size)	Control (size)	Genotype distribution (case/control)					HEW
CC	CT	TT	C	T
*P22 phox gene C242Tpolymorphism and T2DM*											
Hayaishi	2003	Japan	Asian	T2DM (200)	NC (213)	173/182	24/29	3/2	370/393	30/33	0.49
Santos	2005	Brazil	Non-Asian	T2DM (528)	NC (100)	228/47	237/41	63/12	693/135	363/65	0.51
Doi	2005	Japan	Asian	T2DM (130)	NC (490)	103/387	26/97	1/6	232/871	28/109	0.98
Yan	2005	China	Asian	T2DM (137)	NC (68)	125/58	12/10	0/0	262/126	12/10	0.51
Liu	2006	China	Asian	T2DM (194)	NC (105)	154/101	15/2	25/2	323/204	65/6	0.00
Jin	2011	China	Asian	T2DM (186)	NC (139)	42/32	82/70	62/37	166/134	206/144	0.92
Letonja	2012	Slovenia	Non-Asian	T2DM (286)	NC (150)	106/67	138/68	42/15	350/202	222/98	0.71

*P22 phox gene C242T polymorphism and DN*											
Irie	2004	Japan	Asian	DN + T2DM (73)	DN-/T2DM (108)	66/85	6/22	1/1	138/192	8/24	0.94
Santos	2005	Brazil	Non-Asian	DN + T2DM (266)	DN-/T2DM (196)	103/92	126/80	37/24	332/264	200/128	0.51
Narne	2014	Indian	Asian	DN + T2DM (155)	DN-/T2DM (162)	81/88	56/62	18/12	218/238	92/86	0.97
Yan	2005	China	Asian	DN + T2DM (75)	DN-/T2DM (62)	72/53	3/9	0/0	147/115	3/9	0.51
Liu	2006	China	Asian	DN + T2DM (71)	DN-/T2DM (122)	52/101	6/9	13/12	110/211	32/33	0.00
Yang	2006	China	Asian	DN + T2DM (122)	DN-/T2DM (70)	57/49	61/21	4/0	175/119	69/21	0.34
Lim	2006	China	Asian	DN + T2DM (306)	DN-/T2DM (306)	259/268	46/36	1/2	564/572	48/40	0.81

*P22 phox gene C242T polymorphism and CA*											
Hayaishi	2003	Japan	Asian	CA + T2DM (138)	CA-/T2DM (62)	124/49	12/12	2/1	260/110	16/14	0.49
Letonja	2012	Slovenia	Non-Asian	CA + T2DM (254)	CA-/T2DM (32)	96/8	124/16	34/8	316/32	192/32	0.71
Jin	2011	China	Asian	CA + T2DM (94)	CA-/T2DM (179)	14/59	52/89	28/31	66/148	80/120	0.92

T2DM: type 2 diabetes mellitus; NC: normal controls; DN: diabetic nephropathy with T2DM; CA: carotid atherosclerosis with T2DM; DN +/T2DM: T2DM patients with DN; DN-/T2DM: T2DM patients without DN; CA +/T2DM: T2DM patients with CA; DN-/T2DM: T2DM patients without CA; HWE: Hardy–Weinberg equilibrium.

**Table 2:** Results of subgroup analysis and sensitivity analysis.

Category	Sample size (case/control)	T vs. C	TT vs. CC	TT vs. TC + CC	TT + TC vs. CC
OR (95% CI)	OR (95% CI)	OR (95% CI)	OR (95% CI)
*P22 phox gene C242Tpolymorphism and T2DM*					
Overall	1661/1265	1.23 (1.06,1.43)	1.61 (1.14,2.26)	1.50 (1.10,2.05)	1.20 (0.99,1.46)
Asians	847/1015	1.27 (0.74,2.19)	1.87 (0.68,5.13)	1.74 (1.15,2.64)	1.16 (0.67,2.00)
Non-Asian	814/250	1.20 (0.97,1.49)	1.40 (0.86,2.26)	1.26 (0.80,1.99)	1.27 (0.95,1.70)
BH (BO)	1467/1160	1.15 (0.99,1.35)	1.33 (0.93,1.91)	1.30 (0.94,1.79)	1.08 (0.88,1.32)

*P22 phox gene C242T polymorphism and DN*					
Overall	1068/1026	1.25 (1.06,1.47)	1.61(1.08,2.38)	1.45 (0.99,2.12)	1.26 (1.03,1.54)
Asians	802/830	1.14 (0.74,1.76)	1.78 (1.04,3.07)	1.75 (1.03,2.99)	1.08 (0.64,1.82)
Non-Asian	266/196	1.24 (0.94,1.63)	1.38 (0.77,2.47)	1.16 (0.67,2.01)	1.40 (0.96,2.03)
BH (BO)	997/904	1.11 (0.78,1.58)	1.46 (0.93,2.29)	1.31 (0.85,2.01)	1.08 (0.69,1.71)

BH: based on HWE (studies without HWE were excluded); BO: based on the outlier values of OR (studies with the outlier values of OR (OR > 3) were excluded).
